# Evaluation of the Mechanical and Thermal Properties Decay of PHBV/Sisal and PLA/Sisal Biocomposites at Different Recycle Steps

**DOI:** 10.3390/polym11091477

**Published:** 2019-09-10

**Authors:** Alberto Lagazzo, Cristina Moliner, Barbara Bosio, Rodolfo Botter, Elisabetta Arato

**Affiliations:** Department of Civil, Chemical and Environmental Engineering (DICCA), University of Genoa, via all’Opera Pia 15, 16145 Genova, Italy

**Keywords:** biocomposites, PLA, PHBV, sisal, recycling, calorimetry, mechanical testing

## Abstract

The recyclability of polylactide acid (PLA) and poly (3-hydroxybutyrate-*co*-3-hydroxyvalerate (PHBV)-based biocomposites (10%, 20% and 30% by weight of sisal natural fibre) was evaluated in this work. The mechanical and thermal properties were initially determined and were shown to be similar to commodity plastics, such as polyethylene or polypropylene. Three recycle steps were carried out and the mechanical and thermal properties of recycled samples were evaluated and compared to the reference samples. The tensile modulus increased for recycled PLA biocomposites, whereas it was hardly influenced by recycling the PHBV biocomposites. The tensile strength and deformation at the break decreased notably after the first cycle in all cases. Although all the biocomposites became more brittle with recycling, the properties were conserved along until the third cycle, proving their promising recyclability. From the data obtained from the dynamic mechanical analysis, a slight decrease of the storage modulus of PHBV was observed, whereas PLA showed a significant decay of its properties at the 3rd recyclate. The PLA specimens were filled with sisal fibres until they reached 20%wt, which seemed also less subject to the embrittlement occurring along the recycling phase. The characteristic temperatures (glass transition-*T_g_*, crystallization-*T_c_*, melting-*T_m_*) of all the biocomposites were not highly affected by recycling. Only a slight decrease on the melting point of the recycled PHBV was observed suggesting an overall good reprocessability. Moreover, the processing conditions lied in the same range as the conventional plastics which would facilitate potential joint valorization techniques.

## 1. Introduction

Plastics are used for many varied applications in packaging, ranging from sterile storage of medical and pharmaceutical goods, to extending the shelf life of food and protecting sensitive products from damage. Polymers, like poly (ethylene terephthalate) (PET) or polyvinyl chloride (PVC), have a wide, extended use due to their excellent mechanical, chemical and thermal properties. However, their non-renewable nature and lower recyclability are their main disadvantages that need to be dealt with.

Biodegradable polymer materials have been proposed as potential and suitable replacements for traditional plastics as they present good processing properties and a much lower environmental impact [1]. Among all the biodegradable polymers produced from renewable resources, polyhydroxyalkanoates (PHAs) have a very wide range of properties and applications [2,3]. They are synthesized intracellularly by bacteria from agricultural raw materials as a source of carbon and energy. Poly (3-hydroxybutyrate-*co*-3-hydroxyvalerate) (PHBV) is among the most popular of the PHAs [4]. This thermoplastic polyester mainly consists of hydroxybutyrate (HB), along with hydroxyvalerate (HV) units randomly repeated throughout the polymer chain. The physical properties of PHBV vary with the increase of the HV content, which can be controlled by the carbon source supplied during biosynthesis [5]. Among its properties, PHAs exhibit intermediate oxygen and water barrier properties compared to some petroleum based polymers and feature viscosity properties suitable for the melt processes, such as injection, moulding or extrusion [6].

Similarly, polylactide (PLA) is an aliphatic polyester that can be obtained from agricultural resources as corn starch or sugarcane. Its synthesis is a multistep process which starts from the production of lactic acid, with an intermediate step with the formation of lactide and ending with the polymerisation reaction [7]. Currently, PLA is the first commercially used polymer produced from renewable resources [8]. It is biodegradable, recyclable and compostable [9]. Furthermore, it is easily processable on standard equipment used for traditional plastics with a better thermal processability with respect to other biodegradable polymers [10]. It has, however, some disadvantages, such as poor toughness, slow degradation rates, a hydrophobic character and the lack of reactive side-chain groups [11].

The pristine PLA and PHBV can be blended with natural fibres to improve their weak mechanical properties and extend their range of applications [3]. These fibres are strong, light in weight, abundant, non-abrasive, non-hazardous and inexpensive and act as excellent reinforcing agents for bioplastics [12]. Lignocellulosic fibres like jute, sisal, coir, and pineapple have been used as reinforcements in polymer matrices. Among them, sisal is of particular interest as it provides high impact strength besides having moderate tensile and flexural properties compared to other lignocellulosic fibres [13]. Sisal has been used as reinforcement for high density polyethylene [14], polypropylene [15] and polyester [16] among others.

Overall, bioplastics account for nearly 300,000 metric tons of the plastic market (1% of the 181 million metric tons of synthetic plastics produced worldwide) [11]. PLA is the leader in the biodegradable polymers production market with a share of 5.1% in the total production of 5778 million tons in 2016, followed by 2.5% accounted for by PHA biodegradable polymers [17]. This market is growing by 20–30% each year [18] and, following this trend, polymeric waste is also expected to potentially increase. Consequently, its management, once their end of lifetime use is achieved, must be evaluated [19].

Among the different end-of-life scenarios, recycling should be the first option in a circular economy strategy. In most cases, recycled products lose their quality with respect to the initial material. The compliance of the standards established by traditional fossil-derived plastics is crucial to ensure the applicability of biodegradable polymers and their recycled products in an industrial level [20]. The mechanical recycling of biodegradable polymers has been mainly reported for poly(hydroxybutyrate) [21], poly(caprolactone) [22] and poly(lactide) [23,24]. Furthermore, recycling of blends with polycarbonate or HDPE, or biodegradable polymers, such as starch, have been studied [25,26]. A slight decrease of the mechanical properties of pristine PHBV [27] and PLA [28,29] have been shown after several cycles as a result of successive melting or extrusion processing.

Biodegradable polymers can, after a long useful life and after being recycled a maximum number of times, be used in energy recovery systems (i.e., thermal recycling). The generation of heat (or electricity) by incineration is currently the most applied option in Europe [17]. The study of the thermal decomposition of materials stands out as the main basis for the correct tuning of facilities for energy recovery purposes. In this sense, PLA/sisal and PHBV/sisal have proved their suitable behaviour for thermal recycling [30,31].

Within this framework, the main aim of this work is to study the mechanical recycling process of pure polylactid acid (PLA) and poly(3-hydroxybutyrate-*co*-3-hydroxyvalerate) (PHBV) and their sisal-reinforced biocomposites. The mechanical and thermal properties of the new samples at different recycling cycles are discussed and compared with the initial materials in order to ascertain the maximum recycling steps that biocomposites could undergo without losing their main mechanical and thermal properties.

## 2. Materials and Methods

### 2.1. Materials and Sample Preparation

#### 2.1.1. Raw Materials

Poly(3-hydroxybutyrate-*co*-3-hydroxyvalerate) (PHBV) ENMAT Y1000P was obtained from Tianan Biologic (Ningbo, China). Polylactide (PLA) 3251D was purchased from Natureworks (Minnetonka, MN, USA). The sisal fibre was supplied by Thai Royal project (Songkhla, Thailand). The processing of the initial biocomposites was detailed in a previous work [20]. Shortly, the initial samples (R0) were processed to obtain biocomposites with up to 30% in weight of sisal fibres and were labelled as PHBV10/ PLA10, PHBV20/ PHBV30 and PLA10/PLA20/PLA30, where the number indicates the weight percentage of sisal in the formulation. All the materials were mixed in an internal mixer (Brabender PL-2000 Plasti-Corder. Duisburg, Germany) for 5 min at 180 °C and 50 rpm of speed and subsequently pressed by compression moulding (PressesLabPro 400 Press, Fontijne Grotnes. Barendrecht, The Netherlands) into 0.5 mm thick plates, and used as specimens.

#### 2.1.2. Recycled Materials

**Figure 1:**
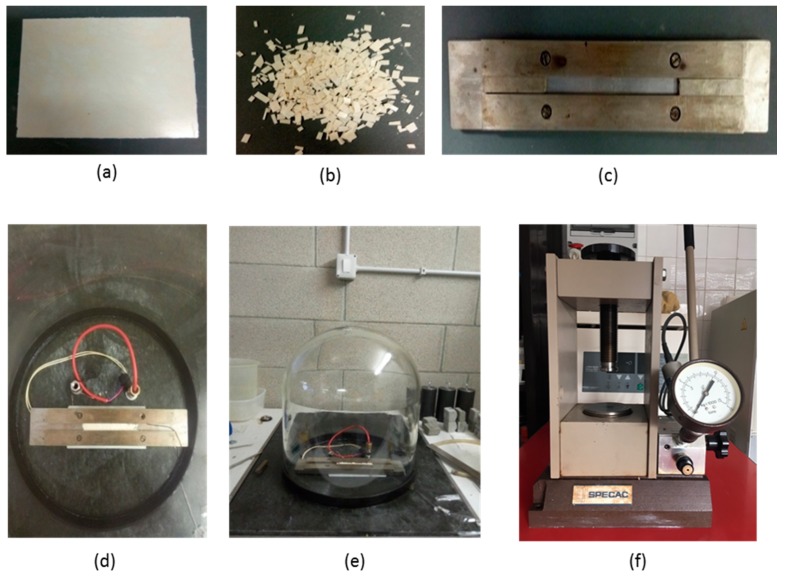
Experimental procedure for mechanical recycling: Raw (**a**) and cut (**b**) material; mold (**c**); resistance (**d**); vacumm (**e**); hydraulic press (**f**).

Three mechanical recycling processes (R1, R2, R3) were carried out on all samples. The recycling scheme is shown in Figure 1. The biocomposites (Figure 1a) were cut into pieces (Figure 1b) to favour the heat transfer phenomena and placed in a rectangular steel mold with dimensions of 15 cm × 1 cm (Figure 1c). The mold was then placed on a rectangular resistance (Figure 1d) with dimensions of 5 cm × 10 cm providing 200 W by a PID (Proportional-Integral-Derivative) controller. The system was placed under a glass cover (diameter = 30 cm; height = 40 cm) (Figure 1e) on a flat surface with a centered orifice and connected to a vacuum pump. The material was carried at the temperatures of 210 °C for PHBV and 220 °C for PLA under vacuum conditions to prevent possible oxidation reactions that can occur during the heating process. Once melted, the samples were cooled until a temperature of 190 °C was reached which took approximately 2 min. Then, they were taken out from the vacuum and manually mixed with a thin spatula, to prevent potential heterogeneities. Finally, when the temperature reached the value of 150 °C, after approximately 5 min, the sample was compressed in a hydraulic press in the air at atmospheric pressure by applying a load of 50 kg, corresponding of a pressure of 0.5 MPa (Figure 1f). This method, although with its limitations and unsuitability for an industrial process, was chosen because of its applicability in a laboratory to obtain recycled panels of PLA and PHBV in order to carry out a preliminary study on the mechanical and thermal properties which lead to the decay of PHBV/sisal and PLA/sisal biocomposites. 

### 2.2. Mechanical Analysis

#### 2.2.1. Static Measurements

The tensile tests and dynamic three points bending test were carried out on a Zwick/Roell Z0.5 electromechanical machine (Zwick Roell Group, Ulm, Germany), using the TesteXpert II software. For both the tests, the rectangular samples of approximately 10 mm in width and 50 mm in length and with a thickness of 0.5 mm were used. In the tensile measurement, the machine was equipped with a vice grip. The tests were run at room temperature, until the sample broke, starting from a distance between the grips of 30 mm. The elastic tensile modulus and ultimate strength were obtained with a constant rate of extension set at 1 mm/min.

For the bending test, a support with two roller pins, of which one was cross tilting, had a span of 30 mm and a with a movable cross tilting loading pin. The flexural modulus of elasticity was obtained with a bending rate of 1 mm/min.

#### 2.2.2. Dynamic Measurements

**Figure 2:**
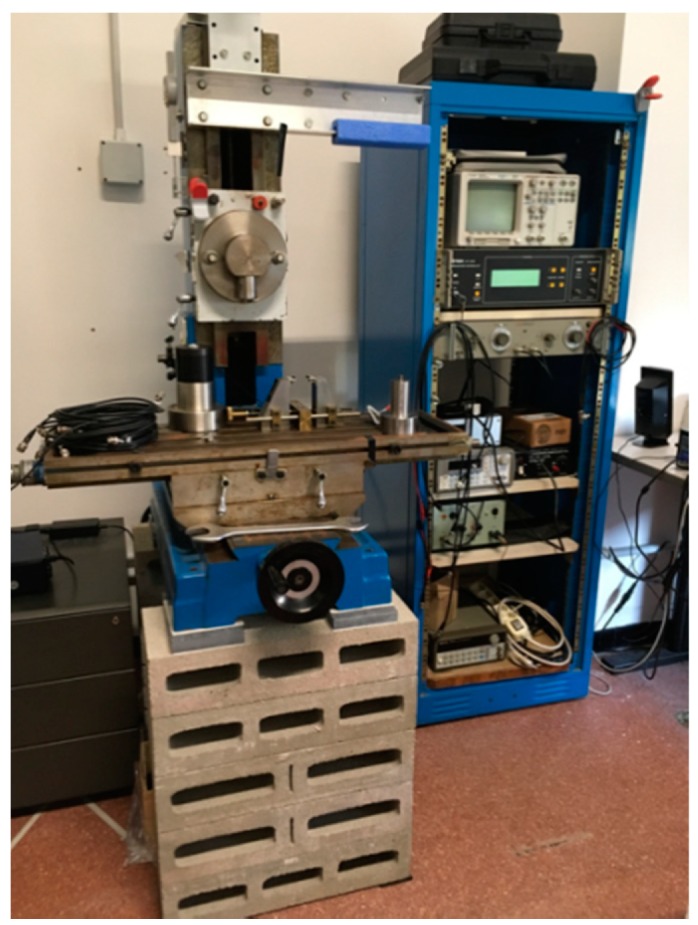
Three points bending apparatus.

The dynamic three points bending test was performed with an apparatus developed in the DICCA laboratory (Figure 2), consisting in six different components: A metallic fixture with two roller supporting pins and a span of 30 mm, placed in contact with an electromagnetic shaker working in the range 1–10000 Hz; a force transducer connected with a roller loading pin to measure the dynamic forces applied on the sample as a consequence of the oscillating movement; a PCB signal amplifier of the force signal; a laser vibrometer, focused on the samples to measure the displacement of the samples; and a National Instrument acquisition card at 32 bits. The signals of the stimulus and response were driven through a Lab View software v. 8.5 and elaborated with fast Fourier transform, obtaining the value of the storage modulus (real part), related to the elastic component of the material and of the loss modulus (imaginary part), and related to the viscous component of the material. Further information on the experimental apparatus can be found elsewhere [32,33].

The tests were performed at room temperature in the range of 1–100 Hz with two different pre-deformations of 0.5 mm and 1.0 mm, respectively. This way, the mechanical behavior could be evaluated at low frequencies (setting corresponding to operative conditions) and high frequencies (as during an impact), all at ambient temperatures.

The maximum dynamic oscillation was calculated equal to 0.15 mm, with a corresponding dynamic force applied ranging between 0.5 N for samples PHBV and PLA, and 1.2 N for samples with PHBV30 and PLA30.

### 2.3. Thermal Analysis

#### Calorimetric Analysis

The tests were carried out using a Calvet Setaram C80 calorimeter (Setaram Instrumentation, Caluire, France) with standard calorimetric cells. The samples of 70 mg, obtained from slices of 0.5 mm in thickness and chopped in pieces of 2–3 mm to assure a more efficient heat diffusion, were analyzed according to a dynamic process with a constant heating rate of 0.2 °C/min, in a temperature range from 30 to 240 °C, in static air. The reference cell was filled with an equal quantity of calcinated kaolin in powder. The error in the measured heat flow was ±5%. The glass transition, crystallization and melting temperatures could be appreciated in the thermograms along the first heating scan [34].

Each of the described experimental analysis was repeated three times and the average was taken as a representative value in all cases.

## 3. Results

### 3.1. Evaluation of Initial Biocomposites Properties

The mechanical and thermal characterization of PHBV and PLA biocomposites was first performed in order to quantify the initial reference properties for all materials. The tensile, dynamic and thermal tests were carried out for all samples and the main results are described in the following sections.

#### 3.1.1. Tensile Tests

**Figure 3:**
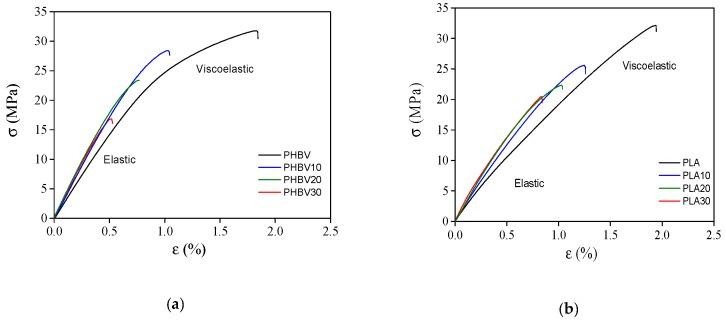
Stress-strain plots for poly (3-hydroxybutyrate-*co*-3-hydroxyvalerate (PHBV) (**a**) and polylactide acid (PLA) (**b**) biocomposites.

Figure 3 shows the stress-strain plots for unrecycled PHBV (a) and PLA (b) biocomposites. In general, an initial linear elastic region is present and its slope increases with the addition of the sisal fibres. Over a yield point, the plot displays a non-linear behaviour, which is more evident in the PHPV samples, and corresponding to a viscoelastic deformation. The presence of the fibres reduces the plasticity of the material considerably.

Table 1 shows the elastic modulus (*E_t_*), tensile strength (*σ_M_*) and elongation at break (*ε_M_*) for all biocomposites. The addition of sisal to the polymeric matrix resulted in an increase in *E_t_* of the reinforced biocomposites, with a maximum increase of 25% for PHBV and 40% for PLA at the highest percentage of fibres. The increased modulus of the composites can be ascribed to a transfer of stress from the matrix to the fibres due to a good wettability between the fibres and the polymeric matrix and then to a high interaction between the two solid phases. These results are in accordance with previous works using other natural fibres as reinforcing agents. For PHBV, the addition of cellulose nanocrystal/silver [35], abaca or jute [3] among others had shown increased elastic modulus values with respect to the pristine biodegradable polymer, as well as bamboo fibres [36], banana fibres [37] or coconut fibres [38] for PLA.

On the contrary, σ_M_ decreased considerably with the increased addition of natural fibres, in particular for PHBV samples (−40%). This fact is probably caused by the addition of extra defects associated to the added short fibres (length 0.5 mm) [39]. Moreover, the low values of ε_M_ evidenced a notable embrittlement of the samples. This implies that the addition of sisal fibres has a negative effect on the toughness of the biocomposites, which has also been shown in other sisal-based composites, such as polyethylene/sisal [14].

Overall, PLA-based biocomposites showed lower elastic modulus (and thus improved stiffness), similar tensile modulus and slightly lower elongation at the break and consequently, lower ductility with respect to PHBV.

These values are comparable to those of traditional thermoplastics. Nearly all the tested biocomposites presented higher elastic modulus than PET (2.8–4.1 GPa), PS (2.3–3.3 GPa) or PP (1.7 GPa). However, these plastics can absorb higher strain before breaking as measured by the elongation at break: PET (30–300%), PS (2.50%) or PP (400%). Finally, the tensile strength of the tested biocomposites differed from PET (48–72 MPa) and were in the same range as PS (34–50 MPa) or PP (38 MPa) [40,41].

#### 3.1.2. Dynamic Tests

**Figure 4:**
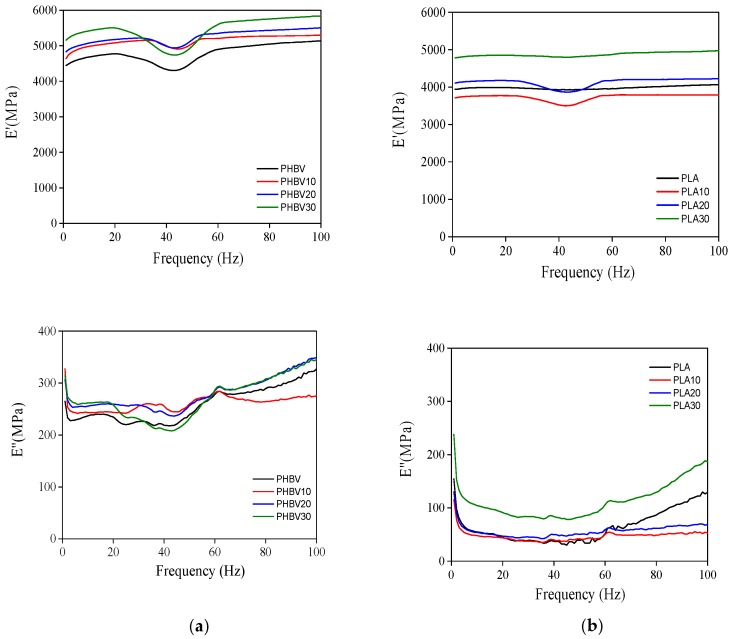
Storage modulus (*E′*) and loss modulus (*E″*) at different frequencies for PHBV (**a**) and PLA (**b**) biocomposites at room temperature (Standard deviation for E’: ±200 MPa for reinforced samples and ±25 MPa for pristine samples; Standard deviation for *E*’’: ±2 MPa).

Figure 4 shows the storage modulus (*E′*) and loss modulus (*E″*) at different frequencies for PHBV (a) and PLA (b) biocomposites at room temperature. The plots show a quite constant trend of *E′* in the range of the explored frequencies. A decrease at 45 Hz for all the PHBV specimens and for PLA10 and PLA20, is clearly observed. This behavior might be due to a resonance effect [42]. In any case, the *E’* data at 20 Hz were taken as representative values, in a frequency region where *E*′ and *E*″ present a constant trend.

Compared to neat PHBV and PLA, the dynamic mechanical properties of the reinforced biodegradable polymers with sisal fibres were enhanced in all cases. The PLA biocomposites showed lower values of *E*′ with respect to PHBV in accordance to other works where pristine PHBV presented the highest storage modulus among all the tested materials [43]. The biocomposites lost deformability and became stiffer, in accordance to what previously observed in the tensile test. The higher standard deviation of the E′ in the reinforced samples (200 MPa) is due to the presence of fibres randomly distributed in the matrix that produce specimens with a lower degree of homogeneity with respect to the samples without fibres (st. dev. 25 MPa). The loss modulus showed a constant trend with the frequency in PHBV samples which increased slightly over 50 Hz, and did not display noticeable differences among the samples with and without fibres. In the PLA specimens, instead, *E*″ remained constant only for PLA10 and PLA20, while a remarkable increase over 50 Hz was observable in PLA and PLA30, the latter doubling the value with respect to the other samples.

**Figure 5:**
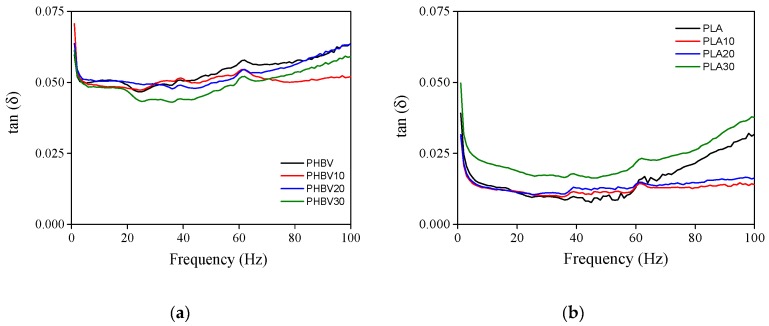
The variation of the loss factor with the frequency for PHBV (**a**) and PLA (**b**) biocomposites.

The loss factor, tan (δ), defined as the ratio of the loss factor (*E*″) to the storage modulus (*E*′), is represented in Figure 5 for PHBV (a) and PLA (b) biocomposites. A constant value at 0.05–0.06 was observed for all the PHBV-based samples while it decreased to 0.01 for PLA, PLA10 and PLA20 and 0.02 for PLA30. The loss factor is used to express the energy dissipated and it provides an indication on the elasticity of the samples and the mobility of the molecular chains. In this case, the higher tan (δ) of PHBV indicated a higher non-elastic strain component in comparison with PLA, thus suggesting a higher mobility of its molecular chains [44].

#### 3.1.3. Thermal Tests

**Figure 6:**
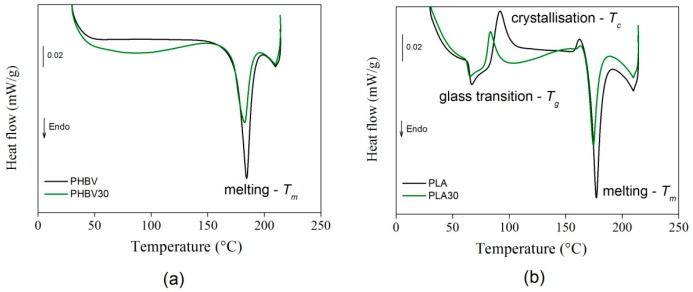
Calorimetric flow curves for PHBV and PHBV30 (**a**) and PLA and PLA30 (**b**) (average error = ±5%).

Figure 6 shows the calorimetric flow curves relative to PHBV and PHBV30 (a) and PLA and PLA30 (b). The cold crystallization and melting temperature were determined from their peaks in the thermograms, and the associated enthalpies (Δ*H_c_* and Δ*H_m_*) were determined using constant integration limits. The PHBV samples displayed a stable behavior until the melting temperature at 184 °C, which was slightly higher than those reported in other works at approximately 170 °C [45]. The presence of sisal fibres at 30%wt seemed to produce only a quite negligible reduction of the melting temperature. The PLA samples instead showed an endothermic peak at approximately 65 °C and an exothermic peak at 92 °C which shifted to a lower temperature (83 °C) in the presence of sisal fibres at 30%wt. In this case, the melting temperature was approximately 170 °C and the addition of fibres did not seem to have an influence on it.

In addition, the degree of crystallinity (*X_c_*) was calculated according to Equation (1) [46]:

(1) $$X_{C}=\frac{\Delta H_{m}}{\Delta H_{mref}\cdot \left(1-x\right)}$$

where $\Delta H_{mref}$ is the melting enthalpy of the 100% crystalline PLA (93.7 J/g) [47] and PHBV (146 J/g) [48] and *x* is the mass fraction of the sisal fiber.

Table 2 presents all the calculated values.

### 3.2. Evaluation of Recycled Biocomposites Properties

The biocomposites were submitted to the mechanical recycling process described in Section 2. The same mechanical and thermal properties described in Section 3.1 for the reference materials were performed on the recycled samples.

#### 3.2.1. Tensile Tests

The tensile tests on the recycled samples were affected by the difficulty to grasp the specimen without inducing damage in the sample due to its fragility. This fact caused a sliding of the sample inside the clamps and a false increase of the measured strain. Moreover, the non-homogeneity of the sample and the possible presence of micro bubbles in the bulk, acting as a crack trigger, reduced the ultimate strength and then the toughness of the material. This effect had also been observed by Mieck et al. for 35% kenaf/PLA composites [49] and also in [50] for kenaf and lyocell fibres.

**Figure 7:**
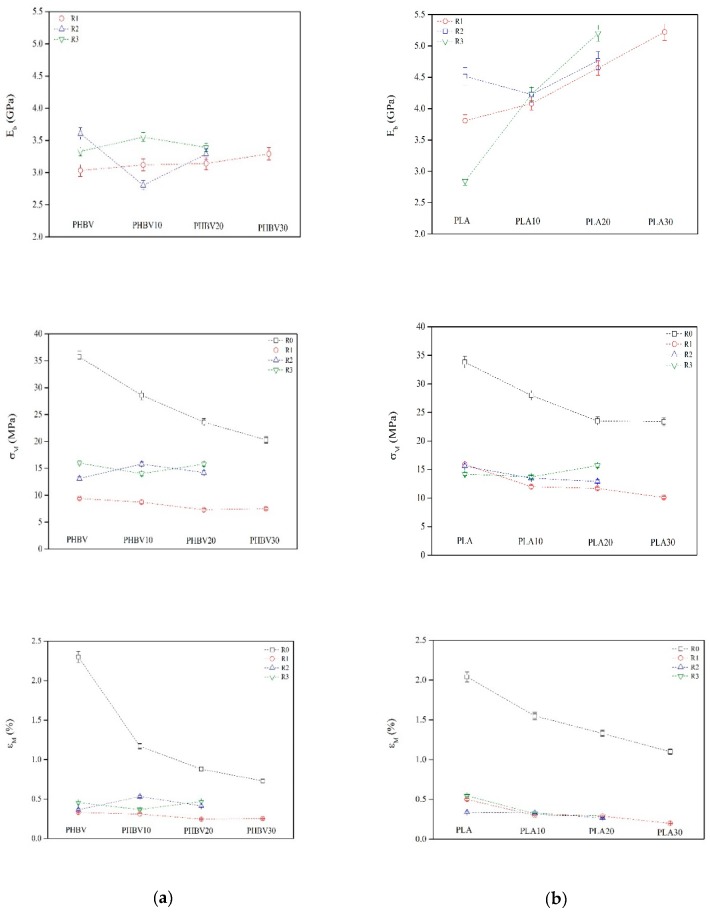
The variation of the elastic flexural modulus (*E_b_*), the tensile strength (*σ_M_*) and the elongation at the break (*ε_M_*) for unrecycled (R0) recycled (R1, R2, R3) PHBV (**a**) and PLA (**b**) biocomposites.

The high variability of the results due to the experimental difficulties led to less reliable values (Table S1 in the Supplementary Material) and flexural tests were performed instead to assess the mechanical properties of the samples. The variation of the elastic flexural modulus (*E_b_*), the tensile strength (*σ_M_*) and the elongation at the break (*ε_M_*) with the recycling steps for PHBV and PLA biocomposites are reported in Figure 7.

An increase on *E_b_* could be observed on both biocomposites after the first and second cycle, whereas a successive slight decrease was observed after the third cycle. The presence of fibres in recycled PHBV and PLA biocomposites did not induce significant differences on *E_b,_* that remained relatively constant after the three recycle steps. In comparative terms, PLA biocomposites presented higher *E_b_* values with respect to those for PHBV indicating an improved stiffness for PLA. These results are in good agreement with those recently reported in the literature for raw materials (PHBV: [27], PLA: [51]; and reinforced biocomposites [52,53]) where no significant differences in the mechanical properties of the recycled samples were found.

The *σ_M_* of the unrecycled biocomposites decreased considerably after the three recycling steps for both types of biocomposites. The recycling process might induce extra defects on the polymeric matrix as a consequence of the random disposition of fibres in the melt leading, thus to a lower cohesion in the material and a lower resistance to break. However, the multiple processing steps did not show great variations on the *σ_M_* revealing a retention on the mechanical properties of the recycled biocomposites during the reprocessing cycles.

The *ε_M_* also decreased with the number of cycles in all cases. The initial values showed unrecycled PLA as the most brittle material but, after three recycling steps, a contrary trend was observed with PHBV presenting lower deformation values. The addition of fibres did not show any effect as all the recycled samples lied in the same range as the deformation values.

In summary, the elastic flexural modulus was hardly influenced by recycling for PHBV biocomposites, whereas it increased for PLA biocomposites. The tensile strength and deformation at the break decreased notably after the first recycling step. Although all the biocomposites became more brittle with recycling, their properties were conserved along until the third cycle indicating a promising recyclability of these materials.

#### 3.2.2. Dynamic Tests

**Figure 8:**
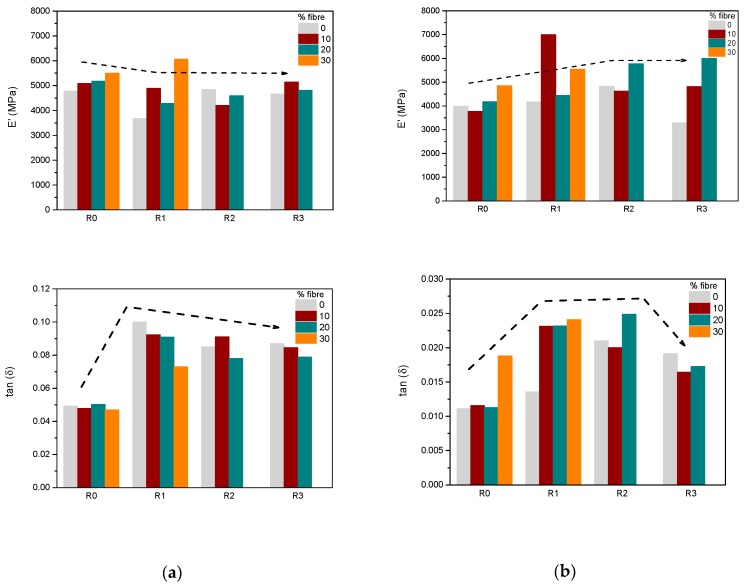
Comparison of the storage modulus (*E’*) and of the loss factor (*tan* *δ*) for PHBV (**a**) and PLA biocomposites (**b**), before recycle (R0) and at different recycles steps (R1, R2 and R3). (Standard deviation for E’: ±200 MPa for reinforced samples and ±25 MPa for pristine samples).

Figure 8 shows the storage modulus (*E*′) and the loss factor (*tan* *δ)* for PHBV (a) and PLA biocomposites (b), before recycling (R0) and at different recycling steps (R1, R2 and R3). In general terms, the storage modulus of PHBV samples did not seem to be highly affected by recycling with a slight decrease of *E’* observed for the recycled PHBV with respect to the unrecycled sample (Figure 8a). For the case of PLA, the storage modulus, *E’*, of the unrecycled biodegradable polymer showed an initial increase with recycling until a final decrease after the 3rd recycle (Figure 8b) which might be explained by the starting of decay of the biocomposites. This deterioration did not occur in the presence of sisal until 20%wt. indicating a positive effect of the fibres to retain elasticity. For both of the polymers, the second and third recyclate over 20%wt. of the fibres could not be performed as it was impossible to produce regular and homogenous samples.

An abrupt increase of the loss factor in the first recyclate followed by a slight decreasing trend are shown for the PHBV and PLA specimens with and without fibres. Overall, a greater loss factor for the recycled samples was found in all cases compared with the unrecycled biocomposites. This behavior might suggest an initial reduction of the brittleness of the material followed by a progressive hardening of the specimens with the recycle steps.

#### 3.2.3. Thermal Tests

**Figure 9:**
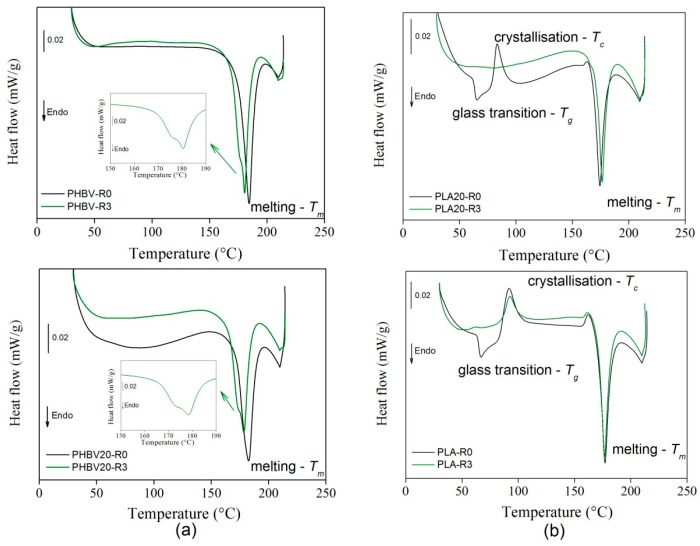
Calorimetric curves for recycled (R3) PHBV and PHBV20 (**a**) and PLA and PLA20 (**b**) and comparison with unrecycled samples (R0).

A previous thermogravimetric analysis [30,31] showed that the trigger of decomposition happened far above their melting point, which ensured a good performance of the composites at high processing temperatures. Figure 9 shows the calorimetric analysis of the samples submitted to recycling. In the specimens containing PHBV, only a reduction of the melting point from 184 °C to 178 °C, respectively for the samples not recycled and after recycling occurred, no glass transition could be observed in the calorimetric signal. Remarkably, the samples with the highest percentage of fibres showed a change of shape from a unimodal to a bimodal distribution (see inset in Figure 9a). This could suggest the existence of a melting-crystallization-melting process in which less perfect crystals melt at lower temperatures, reorganize in more stable crystals during heating and then melt at higher temperatures [51].

**Figure 10:**
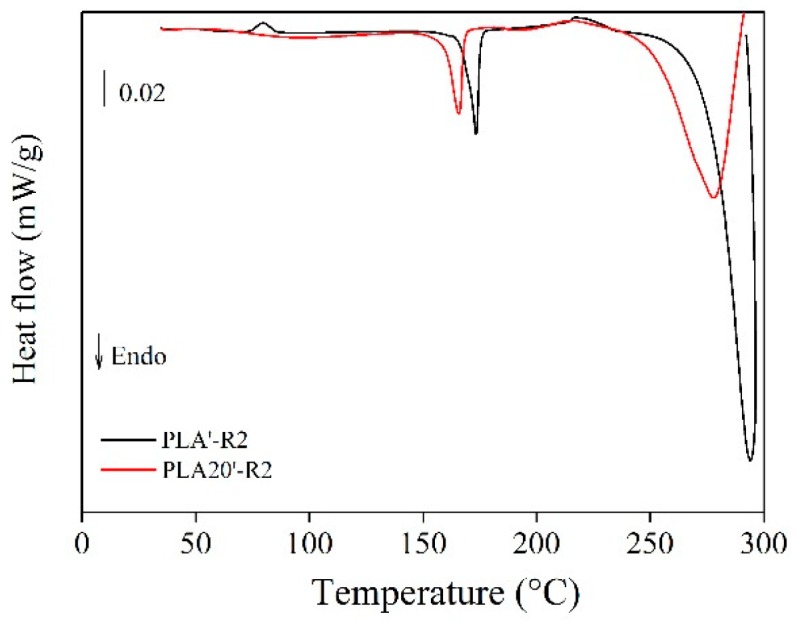
Calorimetric curves for new recycled (R2) PLA’-R2 and PLA20′-R2 samples.

The PLA without fibres (Figure 9b) showed a sensitive reduction of the glass transition (*T_g_*) signal present at 65 °C, which at the 3th recycle became quite negligible. Previous works have observed that this phenomenon is due to a higher molecular chain mobility as a consequence of chain scissions mechanisms and a reduction of fibres geometry during reprocessing [28]. A translation of the exothermic peak corresponding to the crystallization exothermic reaction (*T_c_*) from 95 °C to 99 °C could be observed only at the first recycle. This has been attributed in previous studies to a higher mobility of polymer chains [23]. The melting (*T_m_*) occurred at 177 °C and seemingly, did not depend on the recycling, which is also in accordance with previous works [23,51]. The glass transition and crystallization, instead, disappeared completely in the recycled samples. This phenomenum is probably due to the previously cited microstructural change in the polymeric matrix which interacts with the shorter sisal fibres [28]. In order to assess the replicability of the process, calorimetric tests on new recycled PLA and PLA20 samples (two recycling cycles) were performed up to 300 °C (Figure 10). A sharp endothermic peak is shown due to the polymer decomposition at 293 °C for PLA-R2, which was shifted to 278 °C and reduced in intensity for PLA20-R2. Furthermore, the disappearance of the crystallization peak and of the glass transition for PLA20 was confirmed.

Table 3 presents the characteristic temperatures and the enthalpies associated with those events for unrecycled (R0) and recycled (R1 and R3) samples.

Overall, the crystallisation and melting temperatures were not drastically influenced by the recycling, suggesting a good reprocessability of polymers and the possibility of extending their life service before their final discard. In addition, the characteristic temperatures of all samples lied within the same range indicating that a joint valorization could be performed.

## 4. Conclusions

The changes induced by reprocessing in terms of the mechanical and thermal properties of PHBV, PLA and their biocomposites were investigated and compared to the unrecycled biocomposites. The mechanical and thermal analysis were performed after each of the three reprocessing cycles to evaluate the potential properties decay along the process.

The tensile modulus increased for PLA biocomposites, whereas it was hardly influenced by recycling for PHBV biocomposites. The tensile strength and deformation at the break decreased notably after the first cycle in all cases. Although all the biocomposites became more brittle with recycling, the properties were conserved along until the third cycle indicating a promising recyclability of these materials. In particular, as evaluated through the dynamic mechanical tests, the samples of PHBV were less affected by the deterioration after the recycling processes than the samples of PLA. For the PLA specimens, it also seemed that the presence of the sisal fibres reduced, at least partially, the embrittlement of the material.

Glass transition, crystallization and melting temperatures were not highly affected by recycling suggesting a good reprocessability of the biocomposites. Moreover, the processing conditions lied in the same range as those for conventional plastics which would facilitate potential joint valorization techniques by adapting already existing recycling units.

## Figures and Tables

**Table 1:** Tensile properties of PHBV, PLA and their biocomposites and standard deviation values.

Sample	*E_t_* (GPa)	*σ_M_* (MPa)	*ε_M_* (%)
PHBV	3.1 (±0.01)	35.7 (±0.11)	2.3 (±0.21)
PHBV10	3.4 (±0.05)	28.6 (±0.01)	1.2 (±0.11)
PHBV20	3.6 (±0.07)	23.6 (±0.03)	0.9 (±0.09)
PHBV30	3.8 (±0.02)	20.3 (±0.17)	0.7 (±0.29)
PLA	2.5 (±0.09)	33.8 (±0.09)	2.0 (±0.11)
PLA10	3.0 (±0.02)	28.0 (±0.04)	1.5 (±0.07)
PLA20	3.1 (±0.05)	23.5 (±0.07)	1.3 (±0.06)
PLA30	3.6 (±0.04)	23.4 (±0.12)	1.1 (±0.24)

**Table 2:** Calorimetric results for PHBV, PLA and their biocomposites–raw materials.

Sample	*T_g_* (°C)	*T_c_* (°C)	Δ*H_c_* (J/g)	*T_m_* (° C)	Δ*H_m_* (J/g)	*X_c_* (%)
PHBV	-	-	-	184	94.4	64.7
PHBV30	-	-	-	182	66.3	64.9
PLA	67	92	−29.3	177	64	68.7
PLA30	65	83	−13.7	174	37.3	57.2

**Table 3:** Calorimetric results for PHBV, PLA and their biocomposites–recycled materials.

Sample	*T_g_* (°C)	*T_c_* (°C)	Δ*H_c_* (J/g)	*T_m_* (°C)	Δ*H_m_* (J/g)	*X_c_* (%)
PHBV-R0	-	-	-	184	94.4	64.4
PHBV-R1	-	-	-	178	97.7	66.6
PHBV-R3	-	-	-	180	94.9	64.7
PHBV20-R0	-	-	-	182	66.3	56.5
PHBV20-R1	-	-	-	179	77.1	65.7
PHBV20-R3	-	-	-	178	79.6	67.9
PLA-R0	66	92	−29.3	177	64	68.7
PLA-R1	-	-	-	176	37.9	40.7
PLA-R3	-	-	-	176	39	41.9
PLA20-R0	65	99	−25.9	177	64.8	87
PLA20-R1	-	91	−22.2	177	64.8	87
PLA20-R3	-	92	−13.7	177	57.5	77.2

## Supplementary Materials

The following are available online at https://www.mdpi.com/2073-4360/11/9/1477/s1, Table S1: Tensile results of recycled biocomposites.

## Abbreviations

PHBV	Poly (3-hydroxybutyrate-*co*-3-hydroxyvalerate)
PLA	Polylactide acid
R0	Unrecycled samples
Ri (i = 1, 2 and 3)	Recycling steps

## Symbols

Crystallization temperature	*T_c_ –* °C
Crystallization enthaply	Δ*H_c_* – J/g
Deformation at break	*ε_M_* – %
Degree of crystallization	*X_c_* – %
Elastic modulus	*E_t_ –* Gpa
Elastic flexural modulus	*E_b_ –* GPa
Glass transition temperature	*T_g_* – °C
Loss factor	*tan (δ)* – /
Loss modulus	*E*″ *–* Mpa
Melting temperature	*T_m_* – °C
Melting enthalpy	Δ*H_m_* – J/g
Storage modulus	*E*′ *–* MPa
Tensile strength	*σ_M_ –* MPa
