# Supplementary material for: Evaluation of the Mechanical and Thermal Properties Decay of PHBV/Sisal and PLA/Sisal Biocomposites at Different Recycle Steps
Source: Polymers (Basel). 2019 Sep 10;11(9):1477. doi: 10.3390/polym11091477 (PMC6780613; doi:10.3390/polym11091477)
Supplement: Supplementary file 1 [file polymers-11-01477-s001.pdf]

Table S1. Tensile properties of all bio-composites at the different recycle steps

Sample	Initial bio-composites			Recycle 1			Recycle 2			Recycle 3		
	E_t	σ_M	ε_M	E_t	σ_M	ε_M	E_t	σ_M	ε_M	E_t	σ_M	ε_M
	GPa	MPa	%	GPa	MPa	%	GPa	MPa	%	GPa	MPa	%
PHBV	3.09	35.7	2.30	0.85	13.6	2.09	1.37	18.0	2.11	0.86	13.2	1.60
PHBV10	3.42	28.6	1.17	1.28	17.4	2.38	1.68	10.6	1.58	1.69	13.8	1.71
PHBV20	3.62	23.6	0.88	0.88	13.5	2.08	1.20	7.9	1.00	1.10	10.3	2.16
PHBV30	3.84	20.3	0.73	0.78	8.3	1.80	-	-	-	-	-	-
PLA	2.55	33.8	2.04	1.26	46.1	4.43	1.41	47.6	4.25	1.15	32.0	3.14
PLA10	2.97	28.0	1.55	0.95	26.7	3.26	1.41	15.8	1.91	0.76	12.2	1.99
PLA20	3.12	23.5	1.34	0.82	15.5	2.68	1.28	23.5	2.50	1.39	23.4	1.88
PLA30	3.57	23.4	1.09	1.07	28.1	3.45	-	-	-	-	-	-
